# Adult-Attention Deficit Hyperactive Disorder Symptoms Seem Not to Influence the Outcome of an Enhanced Agonist Opioid Treatment: A 30-Year Follow-Up

**DOI:** 10.3390/ijerph182010997

**Published:** 2021-10-19

**Authors:** Angelo G. I. Maremmani, Pasqualina Rocco, Filippo Della Rocca, Giulio Perugi, Mario Miccoli, Icro Maremmani

**Affiliations:** 1 North-Western Tuscany Local Health Unit, Department of Psychiatry, Tuscany NHS, Versilia Zone, 55049 Viareggio, Italy; angelo.maremmani@uslnordovest.toscana.it; 2 Association for the Application of Neuroscientific Knowledge to Social Aims (AU-CNS), 55045 Pietrasanta, Italy; 3 PISA-School of Experimental and Clinical Psychiatry, 56100 Pisa, Italy; 4 Drug Addiction Unit, 31033 Castelfranco Veneto, Italy; pasqualina.rocco@aulss2.veneto.it; 5 School of Psychiatry, Department of Clinical and Experimental Psychiatry, University of Pisa, 56100 Pisa, Italy; filippo.dellarocca@yahoo.it; 6 VP Dole Dual Disorder Research Group, 2nd Psychiatric Unit, Department of Clinical and Experimental Psychiatry, University of Pisa, 56100 Pisa, Italy; giulio.perugi@med.unipi.it; 7 Department of Clinical and Experimental Medicine, University of Pisa, 56100 Pisa, Italy; mario.miccoli@unipi.it; 8 Saint Camillus International University of Health and Medical Sciences—UniCamillus, 00131 Rome, Italy; 9 G. De Lisio Institute of Behavioural Sciences, 56100 Pisa, Italy

**Keywords:** agonist opioid treatment outcome, A-ADHD symptomatology, heroin addiction

## Abstract

The role of opioids and opioid medications in ADHD symptoms is still largely understudied. We tested the hypothesis that, in Heroin Use Disorder (HUD), when patients are treated with Agonist Opioid medications (AOT), treatment outcome is associated with the presence of Adult Attention-Deficit/Hyperactive Disorder (A-ADHD) symptomatology. A retrospective cohort study of 130 HUD patients in Castelfranco Veneto, Italy, covering 30 years, was divided into two groups according to the Adult ADHD Self-Report Scale (ASRS) score and compared them using demographic, clinical and pharmacological factors. Survival in treatment was studied by utilizing the available data for leaving treatment and relapsing into addictive behavior and for mortality during treatment as poor primary outcomes. Thirty-five HUD subjects (26.9%) were unlikely to have A-ADHD symptomatology, and 95 (73.1%) were likely to have it. Only current age and co-substance use at treatment entry differed significantly between groups. Censored patients were 29 (82.9%) for HUD patients and 70 (73.9%) for A-ADHD/HUD patients (Mantel-Cox test = 0.66 *p* = 0.415). There were no significant linear trends indicative of a poorer outcome with the presence of A-ADHD after adjustment for demographic, clinical and pharmacological factors. Conclusions: ADHD symptomatology does not seem to exert any influence on the retention in AOT of HUD patients.

## 1. Introduction

Attention Deficit Hyperactivity Disorder (ADHD) is a pervasive neurodevelopmental disorder affecting both children and adults. It is clinically distinguished by improper levels of inattention, impulsivity and/or hyperactivity, especially in children and adolescents. In adults, the disorder is almost invariably accompanied by various internalizing and externalizing psychiatric manifestations [1,2,3,4], confounding its identification and treatment managing [5]. About 50% of the ADHD young adolescents show a reduction of hyperactivity, inattention, and impulsivity when they grow up. In 35% of these cases, ADHD symptomatology remains under the clinical threshold, reducing school performance and personal functioning; last of all, in 15% of patients, the whole syndrome continues in adulthood [6,7] and is usually distinguished by an awful and complicated clinical picture [8].

On the whole, A-ADHD patients show a four to nine times greater prevalence of co-existent mental illnesses [9] and are four times more prone to having any mood disorder, when compared with the remaining adult population [10]. ADHD is widely considered a serious risk factor for developing substance use disorders (SUDs) [11]. SUD is found in over 50% of patients with ADHD. Comorbidity between SUD and ADHD is associated with an earlier age at the onset of drug use and a higher likelihood of polyuse (e.g., alcohol, nicotine, cannabis, amphetamines, cocaine, and opioids) [12,13]. Furthermore, ADHD in individuals with SUD appears to be correlated with a higher odds of suicidality, more hospital admissions, a lower probability of achieving abstinence, and lower treatment compliance rates [14,15,16].

Presently, methylphenidate and amphetamines are among the most effective medications for A-ADHD patients [17]. Consistently with Khantzian theory [18], it has also been postulated that the higher probability of cannabinoid, alcohol and heroin use in ADHD adolescents may be in line to achieve subjective relief in combating the ADHD specific psychopathological characteristics, specifically the need for stimulation, mood dysregulation, which is showed by sensitivity to boredom, and the distress correlated with poor cognitive accomplishment [19].

Lastly, the role of opioids in ADHD symptoms continues to be largely understudied and calls for further studies. In two residential treatment programs, 17% of drug users experienced hyperactivity in childhood. Although patients reported being polysubstance users, opioids were usually the drug of choice in people with a history of hyperactivity [20]. Limited studies have analyzed A-ADHD patients seeking treatment for opioid dependence and even rarer studies have described treatment outcomes. In a sample of 157 opioid dependent patients presenting for agonist opioid treatment, approximately 22% were affected by childhood ADHD but, disappointingly, the study did not evaluate the incidence of ADHD symptoms or the possible correlation of these symptoms with treatment outcome [21]. Including treatment outcome, it has been found that 19% of the opioid abusers presented symptoms of ADHD, but at the one-year follow-up, patients with and without ADHD showed no difference in treatment outcome [22]. Patients with ADHD symptoms were statistically less prone to being very successful in maintaining abstinence at nine months after admission to AOT [23].

Our research group has already demonstrated that the correct treatment with stimulants of dual disorder A-ADHD patients dramatically reduced the use of street stimulants. Still, our results were obtained in subjects who were not heroin users or who had only used heroin in the past [24].

As the whole question of the influence of substance use in the treatment of dual disorder A-ADHD patients is still controversial, the present study aimed to apply testing techniques to determine whether the co-occurrence of A-ADHD symptomatology in HUD patients can influence clinical and long-term therapeutic aspects in an out-patient Agonist Opioid Treatment (AOT) setting. On this topic, we aimed to figure out the clinical impact of opioid medications on ADHD psychopathology.

## 2. Materials and Methods

### 2.1. Setting

We identified people resident in the Northeast part of Italy (Castelfranco Veneto, province of Treviso, Veneto Region) registered at the local drug addiction unit and treated with AOT medications (methadone or buprenorphine) for their heroin addiction for 30 years. This study population represents all individuals attending the local public (service in Italy with a population of approximately 115,000 subjects). We collected demographic details, clinical aspects of Heroin Use Disorder (HUD) and its psychiatric comorbidity, therapeutic medications used and treatment outcomes.

In the Castelfranco Veneto AOT, the Dole and Nyswander (D&N) methodology of treatment [25,26] has been used since its foundation. It involves four broad stages. During the Induction Phase, the patient replaces street heroin with the maintenance medication (generally methadone and/or buprenorphine). The induction phase involves an initially low dose, followed by titration over subsequent days to achieve a stable dose (including the objective of achieving a steady-state plasma concentration). Then the dosage is increased to provide a maintenance blocking dose able to suppress opioid withdrawal signs and symptoms, without producing euphoria. Then the patients are maintained on a stable dosage while supervising treatment evolution and changing the dose if necessary. During this period, psychosocial interventions can be recommended [27]. The latest stage is named “medically supervised tapering”. While remaining in treatment is an important goal, patients should be helped to withdraw from medications. Gradually lowering the dose is the best way to safety withdrawal from the medication.

### 2.2. Assessment

#### 2.2.1. Clinical Global Evaluation (CGI) Rating Scale

The CGI consists of three items. Severity of Illness varies from (0) normal to (7) among the most extremely ill, on a seven-point scale. Global Improvement varies on a similar seven-point scale, from (1) very much improved to (7) very much worse. The Efficacy Index considers the ratio Therapeutic Effect/Side Effect. Therapeutic effect is ranging from (1) Unchanged or Worse to (4) Marked; side effect is ranging from (1) None to (4) Outweighs.

#### 2.2.2. Adult ADHD Self-Report Scale (ASRS)

The frequency of self-reported adult ADHD symptoms, derived from the DSM-IV criteria, was assessed by the ASRS, a Likert scale, ranging from 0 (“never”) to 4 (“very often”). Participants who have a score below 16 are unlikely to have A-ADHD; those in the 16-24 range are likely to have A-ADHD, and those over 24 are highly likely to have an A-ADHD diagnosis.

#### 2.2.3. Other Psychiatric Comorbidities

No formal psychiatric diagnosis was required to be admitted to the program. Independently of recruitment criteria, the clinical records collected dichotomous information from these patients to document: The lifetime presence of any psychiatric diagnosis, previous hospitalization referable to psychiatric reasons; suicide attempts; and mental disability benefits. Additional psychiatric information was extracted from follow-up clinical records, which were compiled during the stay in treatment of the patients, so allowing the collection of data regarding the use of psychiatric medications during the program. We classified patients as having experienced lifetime psychiatric difficulties if they showed one or more of these psychiatry-related conditions; alternatively, if they did not show any such psychiatric proxies, they were classified as patients whose lives had remained free of any other lifetime psychiatric problems.

### 2.3. Data Analysis

We presented data as the number (percentage) of individuals for categorical variables. We divided patients into two groups based on A-ADHD symptoms co-occurrence. Univariate differences were tested through nonparametric tests (comparing medians across groups using the Wilcoxon–Mann–Whitney Z test) for metric variables and Chi-squared analysis (comparing column proportions) for categorial ones, using Fischer’s Exact Test when necessary.

Comparison of retention in treatment was performed using the Kaplan–Meier survival analysis and the Log Rank (Mantel–Cox) test for comparison between groups. For the analysis, positive outcome (‘censored observations’) refers to patients still in treatment or leaving treatment for reasons not related to the treatment itself (e.g., when stabilized patients were moving to other towns/cities). When a patient dropped out of the treatment by not presenting at the scheduled follow-up, we considered it a ‘terminal’ event (negative outcome). For positive outcome, the following characteristics have been added: “if symptoms are present, they are transient, acceptable reactions to psychosocial stressors (e.g., difficulty in concentrating after family arguments): no more than modest damage in social, occupational, or school functioning. In cases that had a ‘negative’ outcome, these criteria were not met.

We assessed survival rates for the patients according to the presence of a comorbid A-ADHD symptomatology. The association between A-ADHD and retention in treatment was abridged using Cox regression (Omnibus Tests of Model Coefficients). Sociodemographic, clinical and symptomatological variables that may act as confounding factors influencing a negative outcome were included in our model (Age; Male sex; A-ADHD/HUD symptomatology presence; Living with a partner; High Education (more than 8 years; Treated with methadone; Unemployed; Only heroin predominant use; Predominant Cocaine/Alcohol co-use; Predominant Cannabinoid co-use; Presence of another psychiatric comorbidity; CGI1 baseline severity; CGI endpoint efficacy index).

In consideration of the exploratory nature of the study, we referred to levels of significance of *p* < 0.05.

## 3. Results

### 3.1. Cohort Characteristics

Data were obtained on all 130 individuals who entered treatment at any time between 14 October 1987, and 19 October 2020; to avoid discrepancies, they were, without exception, evaluated by the same doctor on duty (PR). Table 1 shows the descriptive statistics for the study population. Most of the patients were males, aged around 35 years at the time of the study; they were single, with a low educational level (≤8 years of learning), stably employed, without welfare benefits and living with their original family. At treatment entry, they were predominantly using heroin, alcohol and cocaine, and, considering the entire observational period, had been treated with opioid medications and, to a lower extent, with antidepressants (17.7%), mood stabilizers (8.5%) and major or minor sedatives (32.3%). An additional psychiatric diagnosis was present in 52 (40%) patients.

Most of our cohort were regularly observed for about ten years (within the range the maximum was 33 years). When treatment began, patients were markedly ill. In contrast, at the endpoint, the severity of illness had been reduced so that patients appeared mildly ill, with partial remission of symptoms and no side effects.

According to our assessment, 99 (76.2%) patients showed a good and 31 (23.8%) a poor outcome. More specifically, seven (5.4%) patients successfully completed their program. Twenty-one (16.2%) were transferred to other services as stabilized patients. As many as 71 participants (54.6%) had stayed in treatment. Five (3.8%) patients had died, while 26 (20.0%) patients had dropped out of the program.

According to the ASRS test, our cohort could be divided into two groups. Thirty-five subjects (26.9%) were unlikely to have A-ADHD, whereas 95 (73.1%) were likely to have A-ADHD (A-ADHD/HUD).

### 3.2. Demographic, Clinical and Treatment Outcome Differences between HUD Patients with and without ADHD Symptoms

We found no statistically significant differences between HUD patients with and without A-ADHD symptomatology, the only exceptions being their age at the time of the study and co-substance use (Table 1). The HUD patients most likely to have ADHD were younger and made more frequent use of alcohol and stimulants than their peers.

**Figure 1:**
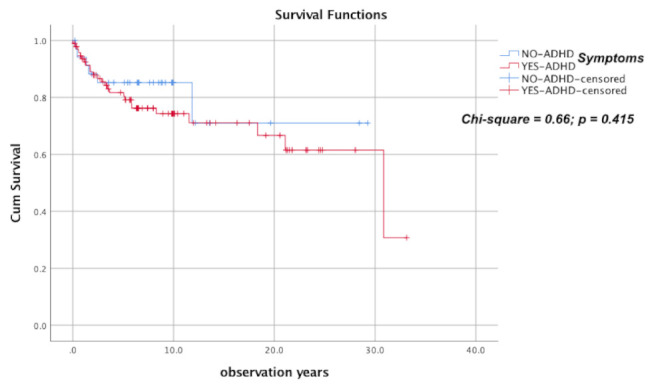
Survival in treatment of 130 HUD patients according to the co-presence of A-ADHD symptoms.

Table 2 shows observed patients and terminal events according to the two groups. The cumulative proportion of surviving patients at the end of the observational period was 0.71 for HUD and 0.30 for A-ADHD/HUD patients; censored patients were 29 (82.9%) for HUD patients and 70 (73.9%) for A-ADHD/HUD patients (Figure 1). This difference was not statistically significant at Mantel-Cox test (Chi-square = 0.66; Sig. 0.415). Following the addition of potential confounding factors (Table 3) in a Cox proportional hazards model, an association was found between retention in treatment and investigated covariates (Chi-squared = 44.22; *p* < 0.001) only regarding patients’ age (HR = 0.93; 95%CI: 0.88–0.98) and CGI endpoint efficacy index (HR = 3.64; 95%CI: 2.03–6.50).

## 4. Discussion

### 4.1. Epidemiology

As many as 73.1% of our HUD patients presented A-ADHD symptomatology—a much higher percentage than those reported in the literature [20,21]. This association finds a credible explanation, as mentioned above, in the self-medication hypothesis (SMH), especially regarding heroin and cocaine. The SMH was first originally developed by Khantzian, who proposed that the drive to use drugs may be by the user as the best solution to the need to relieve distress and suffering [28]. According to Khantzian, there is a large degree of psychopharmacological specificity in the drug(s) selected by each user [29], suggesting that these selections are not chosen casually. They result from an interaction between the drug’s pharmacological action and psychopathological symptoms causing potent distress, compounded with the factors of vulnerability and proneness. In other words, subjects self-select drugs to use according to their personality structure and related disabilities.

Consequently, patients use drugs to reduce ADHD symptoms. Stimulants, including cocaine, can, paradoxically, act to calm and counteract emotional lability, hyperactivity, and inattention in ADHD patients [30]. In one of our studies [24], treating A-ADHD/CUD patients with stimulant medications seemed to improve both ADHD symptomatology and cocaine use. Stimulant medications (i.e., Atomoxetine and Methylphenidate) have proved to act on the mental distress caused by an untreated ADHD [17], thus suggesting that cocaine use could lower the urgent need for relief from the distress caused by ADHD. In other words, we confirmed the idea that the use of cocaine may work as self-medication behavior in subjects who feel less interest in pleasurable feelings and reward than non-CUD individuals who have ADHD. In a psychiatric setting, A-ADHD has correlated to the use of cocaine-alcohol and cannabinoids, and the use of heroin is sporadic [31,32]. In the present study, in a drug addiction unit, the use of heroin seems to be predominant. Many risk factors are common features of ADHD and HUD, in particular impulsivity and sensation-seeking.

Similarly, executive dysfunction and poor judgment may lead individuals with ADHD to try heroin and cocaine, increasing the vulnerability to developing addiction disorders compared to their peers without ADHD. Like HUD patients, ADHD individuals have often reported difficulties in delaying or modulating the reward response, with a strong tendency to develop multiple addictive behaviors [33]. Finally, ADHD and HUD are associated with increased exposure to psychosocial risk factors, including educational failure and easy exposure to drugs. Using cocaine, subjects with ADHD could have relief from inattentiveness, and heroin comes as temporary relief from impulsiveness and dysphoric mood.

### 4.2. Demographic, Clinical and Treatment Outcome Characteristics According to the Presence of ADHD Symptomatology

In summary, at the time of the study, HUD patients likely to have ADHD were younger and, at treatment entry, made more frequent use of alcohol and stimulants than their peers. The retention rate of A-ADHD/HUD failed to reveal any statistically significant differences. Older patients were more frequently retained in treatment than younger ones, and the negative outcome was correlated with the absence of improvement at the most recent clinical evaluation.

HUD patients—considering both those with and without A-ADHD—did not show differences when their investigated demographic data were compared regarding the severity of illness at treatment entry or during the study. They also showed the same retention in treatment and outcomes in dropping out of treatment, successful termination, and treatment continuation. The number of deceased patients or those transferred as stabilized patients revealed no statistical differences. Long-term retention in treatment was no different, and demographic, clinical and therapeutic variables were not correlated with a negative outcome.

In our sample, we found only two variables related to negative outcome. Older patients were more frequently retained in treatment than younger ones, and a negative outcome was correlated with the poor efficacy of treatment at the last clinical evaluation. These two results appear to be tautological when analyzing a long-term treatment.

The first consideration to be made, at this point, is about the use of sedatives (major and minor); in our cohort, the frequency was around 30%, without negative effects on the outcome. Treating A-ADHD with opioids, which can themselves be considered sedative medications, does not seem to have, in our cohort, the same clinical impact as the use of other sedatives, such as benzodiazepines (BDZs). As GABA-agonist drugs, the non-medical use of BDZs and Z-drugs has shown a significant correlation with ADHD. In a way, like alcohol use, from the SMH perspective, BDZ may offer short-term relief for restlessness and hyperactivity in ADHD subjects. Regrettably, long-term and high doses of BDZ have been shown to induce the significant clinical impairment of patients’ neurocognitive and executive functions and quality of life, regardless of whether they have ADHD or not [34,35]. BDZ-induced neurocognitive deficit seems to persist even following the suspension of BDZ [36,37]. It has been suggested that the clinical impact of the long-term use of BDZ may be even higher in ADHD subjects [38]. In addition, in the present study, HUD/A-ADHD patients treated with D&N AOT seem to have results that differ from those obtained in an Italian polycentric study [39]. Regrettably, AOT methodology in Italy is not strictly linked to a D&N methodology, as shown in the use of blocking doses for the long term [40], as in the Castelfranco Unit.

In general, persistent ADHD symptoms can adversely affect many dimensions of an individual’s life, including physical, social, occupational, and behavioral functioning, and can lower the overall quality of life [41,42], but in our opioid-treated patients, no differences were found discriminating patients’ social adjustment between HUD with and without A-ADHD during D&N-AOT. Comorbid ADHD and SUD appear to exacerbate several maladaptive SUD outcomes. Earlier drug use initiation increased psychiatric comorbidities, hospitalizations, suicide attempts, and HIV-risk behaviors [14,43], making the management and treatment of SUD in clinical settings more challenging and less effective [44,45]. Poor treatment compliance, slower SUD remission, and greater risk of relapse have been repeatedly demonstrated in these patients [46], but this does not seem to happen during D&N-AOT.

The second consideration is that the only difference found between groups is that A-ADHD/HUD patients at treatment entry took more stimulants and alcohol than those with HUD but without A-ADHD. In a recent study, by applying a factorial analysis we, clustered our dual disorder A-ADHD patients in two different typologies of substance use and then correlated these typologies with clinical A-ADHD aspects. Two patterns of substance use were identified: The first (type 1) was distinguished by stimulants/alcohol, and the second (type 2) using cannabinoids (THC). Type 1 users were significantly younger and had more legal problems, but the two patterns were similar in terms of ADHD-specific symptomatology and severity at treatment entry [31,32]. Dual disorder A-ADHD patients seem to use less cocaine and alcohol when HUD characterizes dual disorder. In heroin addicts receiving enhanced methadone treatment, we have already demonstrated that both the cessation of illicit opioid abuse and retention in treatment was positively correlated with an attenuation of alcohol and cocaine abuse, together with the absence of the psychosocial complications associated with such abuse. So, it is not surprising that A-ADHD/HUD patients show the same outcome as HUD patients without A-ADHD.

We have already observed a dramatic reduction of cocaine use in dual disorder patients with concomitant ADHD treated with stimulant and non-stimulant A-ADHD medications [24]. We have speculated that our patients with A-ADHD but without HUD may use stimulating drugs to reduce their inattentive ADHD symptoms, aiming to alleviate their attention level. Thus, we defined this cocaine desire as a “relief” craving from the cognitive and emotional distress arising from ADHD, which paved the way for severe impairment in the quality of life while drastically compromising professional and social functionality. This type of craving is different from that usually reported by cocaine users who are free from ADHD. These patients are mainly driven by the recreational/rewarding effects induced by the stimulant. One of our studies suggested that bipolar patients can use stimulants during the manic or hypomanic phase for rewarding reasons [47]. It remains to be explained why, in this study, A-ADHD/HUD patients with the greatest use of cocaine responded to treatment even in the absence of specific medications for A-ADHD.

Interactions between opioid and dopaminergic systems have been documented. Both stimulants and opioids can increase dopamine release in the brain. The former directly enhances the effect of dopamine, and the latter indirectly promotes dopamine levels by activating opioid receptors [48]. From a neurobiological viewpoint, studies are increasingly suggesting that activation of the kappa-opioid receptor (KOR) is involved both in the stress response and in the drug rewarding process. KOR is a G-protein-coupled receptor activated by the endogenous neuropeptide dynorphin [49,50,51,52,53]. Stress-induced activation of the dynorphin-KOR system is associated with dysphoria, analgesia, anxiety, depression, and drug-seeking behaviors [54,55,56,57,58,59,60]. More interestingly, KOR activation has been shown to decrease frontal cortical DA release [61,62], even in the absence of stressful conditions [61,62]. KOR activation also decreases noradrenaline release from presynaptic neurons [63]. On this basis, pre-treatment with KOR antagonists (norbinaltorphimine, norBNI) has been shown to improve cognitive function and reduce frontal cortical DA and noradrenaline in preclinical ADHD-animal models exposed to nicotine in the perinatal period. The effects which have been reported were comparable with those of methylphenidate but were longer-lasting and slower in the onset phase, without producing significant changes in the tissue content of DA in the nucleus accumbens [63]. Pre-treatment with a KOR antagonist (norBNI) also proved to block cocaine place preference conditioning previously induced by repetition of the forced swim test [64]. It is interesting that ADHD/HUD subjects have been treated, in a way involving a higher, though not significant, percentage of their peers without A-ADHD, with buprenorphine, which is a powerful k receptor antagonist.

Lastly, it should be noted that 4-hydroxyatomoxetine, the human metabolite of atomoxetine, is a partial KOR agonist [65]. Subsequently, it is possible to point to KOR antagonists as potential therapeutic agents for ADHD management, particularly buprenorphine [66].

A-ADHD/HUD patients use more cocaine than those with HUD alone, confirming the craving for relief by taking cocaine in these patients too. However, a stimulant such as methylphenidate has been shown to activate the μ opioid receptor (MOPR) in the brain, particularly when taken at sufficiently high doses [67,68,69], suggesting the presence of stimulant relief craving even in decompensated heroin addicts. Proper use of opioid medications could correct imbalances of that kind. Relief craving for stimulants in opioid users is again supported by the fact that chronic opiate users have been associated with reduced densities in norepinenaline (α2) and dopamine (D2) receptors [70,71], but no evidence for neurotoxic effects on dopamine neurons has been identified [72]. In particular, the overall impact on the dopamine system in opiate users are less pronounced than that in stimulant users [72]. Structural and functional abnormalities in opiate users are less specific than those observed in amphetamine users [73,74,75,76,77,78].

Limitations: The obvious limitations of this study are because this is a retrospective analysis carried out on a relatively small cohort of patients rather than a study specifically designed to elucidate this issue. Assessments of the same patient in various clinical presentations of the natural history of this illness would have provided more widespread evidence. A better level of information would be derived from a specific protocol. In addition, the ARS is not sufficient to make a retrospective ADHD diagnosis or to make any consideration on ADHD as a diagnostic category. Since ADHD in adults is a chronic condition that begins in childhood, it is necessary to estimate the symptoms, development and relative level of functional impairment during childhood, using a retrospective interview aimed at investigating the behaviors relating to that period of life. The information collected from the patient should be supplemented by significant people who knew the subject when he was a child (usually parents or close relatives).

This study, however, covers a very long-time span and enables us to study the effect of opioid medications on ADHD symptomatology in the absence of the concomitant use of stimulants for A-ADHD. In Italy, stimulants for A-ADHD can only be prescribed in selected psychiatric centers that cannot prescribe opioid medications. Addiction Units only can prescribe opioid medications; however, these units cannot use stimulant drugs for A-ADHD/HUD patients. This situation was typical of the Castelfranco Veneto Addiction Unit.

## 5. Conclusions

In HUD patients with or without A-ADHD symptomatology, an enhanced AOT designed according to the D&N methodology is beneficial in both cases, as significant changes in the outcome of these patients cannot be highlighted.

## Figures and Tables

**Table 1:** Differential characteristics of Heroin Use Disorder patients according to the co-presence of A-ADHD symptoms.

	Total Sample N = 130	Without A-ADHD Symptoms N = 35	With A-ADHD Symptoms N = 95		
	Median	Median	Median	z	p
Current age	39.00	44.00	39.00	−2.23	0.026
Length of observation years	6.53	7.62	6.47	0.49	0.896
Severity of illness at baseline	4.00	4.00	5.00	−1.76	0.079
Severity of illness at endpoint	3.00	3.00	3.00	−1.87	0.061
Global improvement	2.00	2.00	2.00	−0.39	0.697
Efficacy index (Therapeutic effect/Side Effects)	5.00	5.00	5.00	−0.49	0.627
	N (%)	N (%)	N (%)	χ^2^	p
Gender (Male)	107 (82.3)	30 (85.7)	77 (81.1)	0.38	0.537
Marital Status (Single)	102 (85.4)	27 (77.1)	84 (88.4)	2.60	0.106
Education (High School)	44 (33.8)	16 (45.7)	28 (29.5)	3.01	0.083
Working				5.39	0.220
Unemployed	34 (26.2)	5 (14.3)	29 (30.5)		
Stably employed	71 (60.8)	26 (74.3)	53 (55.8)		
Unstably employed	11 (8.5)	2 (5.7)	9 (9.5)		
Welfare benefit	5 (3.8)	2 (5.7)	3 (3.2)	0.45	0.611
Living situation				1.57	0.667
Origin family	69 (60.8)	17 (48.6)	52 (54.7)		
Procreation family	42 (32.3)	11 (31.4)	31 (32.6)		
Alone	16 (12.3)	6 (17.1)	10 (10.5)		
Community	3 (2.3)	1 (2.9)	2 (2.1)		
Co-substance use				11.85	0.003
Only heroin	44 (33.8)	20 (57.1)a	24 (25.5)b		
Predominant Stimulants-Alcohol	46 (35.4)	7 (20.0)a	39 (41.1)b		
Predominant cannabinoids	40 (30.8)	8 (22.9)a	32 (33.7)a		
Other lifetime psychiatric comorbidities	52 (40.0)	12 (34.3)	40 (42.1)	0.65	0.420
Agonist Opioid Medication				0.75	0.386
Buprenorphine	41 (31.5)	9 (25.7)	32 (33.7)		
Methadone	89 (68.5)	26 (74.3)	63 (66.3)		
Medications used					
Mood stabilizers	11 (8.5%)	2 (5.7)	9 (9.5)	0.46	0.726
Antidepressants	23 (17.7)	3 (8.6)	20 (21.3)	2.73	0.123
Major and minor sedatives	42 (32.3)	11 (31.4)	31 (32.6)	0.17	0.896
Good outcome	99 (76.2)	29 (82.9)	70 (73.7)	1.18	0.276
Treatment outcome				4.52	0.322
Abandon	26 (20.0)	5 (14.3)	21 (22.1)		
Completer	7 (5.4)	3 (8.6)	4 (4.2)		
Still in treatment	71 (54.6)	23 (65.7)	48 (50.5)		
Deceased	5 (3.8)	1 (2.9)	4 (4.2)		
Transferred	31 (16.2)	3 (8.6)	18 (18.9)		

‘a’ and ‘b’ letters denotes letters denote a subset of categories whose column proportions differ significantly from each other at the 0.05 level.

**Table 2:** Observed patients and terminal events according to the time intervals.

	Observed Patients		Terminal Events	
Time Interval (Years)	HUD	HUD/ADHD	HUD	HUD/ADHD
0–5 years	35	95	5	16
6–10 years	26	69	0	5
10–15 years	6	28	1	1
15–20 years	3	18	0	1
20–25 years and beyond	2	15	0	1

**Table 3:** Predictors of negative outcome.

		95.0% CI		
	HR	Lower	Upper	*p*
A-ADHD/HUD presence	1.04	0.39	2.80	0.936
Male sex	0.41	0.16	1.07	0.069
Living with a partner	0.76	0.16	3.51	0.724
High Education (more than 8 years	0.81	0.37	1.79	0.604
Treated with methadone	0.83	0.35	1.96	0.665
Unemployed	0.45	0.17	1.15	0.095
Only heroin predominant use	1.00			0.671
Predominant Cocaine/Alcohol co-use	0.96	0.35	2.59	0.928
Predominant Cannabinoid co-use	1.45	0.52	4.06	0.481
Presence of another psychiatric comorbidity	0.65	0.29	1.46	0.299
CGI1 baseline severity	0.97	0.67	1.41	0.874
CGI endpoint efficacy index	3.64	2.03	6.50	<0.001
Age	0.93	0.88	0.98	0.006

Omnibus Tests of Model Coefficients: Chi-square = 44.20; *p* < 0.001.

## Data Availability

For data availability, please contact the authors.
